# Supplementary material for: Structural and mutational analysis of the ribosome-arresting human XBP1u
Source: eLife. 2019 Jun 27;8:e46267. doi: 10.7554/eLife.46267 (PMC6624018; doi:10.7554/eLife.46267)
Supplement: Supplementary file 1. — Summary of parameters related cryo-EM data collection and processing. [file elife-46267-supp1.docx]

**Supplementary File 1.**

|  | **XBP1-RNC** | **XBP1-RNC** | **XBP1-RNC-SRP** | **XBP1-RNC-Sec61** |
| --- | --- | --- | --- | --- |
| Ribosomal state | Post State | Rotated state | Post state | Post state |
| Microscope | FEI Titan Krios | FEI Titan Krios | FEI Titan Krios | FEI Titan Krios |
| Camera | Falcon II | Falcon II | Falcon II | Falcon II |
| Voltage (kV) | 300 | 300 | 300 | 300 |
| Pixel size (Å) | 1.084 | 1.084 | 1.084 | 1.084 |
| Electron dose (e-/Å^2^) | 28 | 28 | 28 | 28 |
| Defocus range (µm) | 0.5 - 2.5 | 0.5 - 2.5 | 0.5 - 2.5 | 0.5 - 2.5 |
| Particles after 2D (no.) | 531952 | 531952 | 170231 | 43578 |
| Final particles (no.) | 223773 | 94923 | 24875 | 12749 |
| **Model Composition** |  |  |  |  |
| Protein residues | 11717 | 11673 | 12566 | 12239 |
| RNA bases | 5669 | 5797 | 5874 | 5668 |
| **Resolution (Å)** | 3 | 3.1 | 3.7 | 3.9 |
| FSC threshold | 0.143 | 0.143 | 0.143 | 0.143 |
| Map CC (around atoms) | 0.76 | 0.75 | 0.71 | 0.68 |
| Map CC (whole unit cell) | 0.73 | 0.72 | 0.68 | 0.66 |
| Map sharpening B-factor (Å^2^) | -71.2 | -59.9 | -105.54 | -81.6 |
| **RMS Deviations** |  |  |  |  |
| Bond lengths (Å) | 0.004 | 0.0038 | 0.0036 | 0.0035 |
| Bond angles (°) | 0.91 | 0.92 | 0.88 | 0.91 |
| **Validation** |  |  |  |  |
| MolProbity score | 1.5 | 1.66 | 1.55 | 1.5 |
| Clashscore | 4.9 | 4.82 | 5.34 | 4.55 |
| Poor rotamers (%) | 0.23 | 0.20 | 0.16 | 0.13 |
| **Ramachandran Plot** |  |  |  |  |
| Disallowed (%) | 0.03 | 0.09 | 0.05 | 0.02 |
| Allowed (%) | 3.60 | 5.67 | 3.84 | 3.87 |
| Favored (%) | 96.37 | 94.24 | 96.11 | 96.1 |
