# Supplementary material for: Structural and mutational analysis of the ribosome-arresting human XBP1u
Source: eLife. 2019 Jun 27;8:e46267. doi: 10.7554/eLife.46267 (PMC6624018; doi:10.7554/eLife.46267)
Supplement: Supplementary file 2. — Representing red curve (mutation S255A in the XBP1u AP) and blue curve (mutations S255A and P254C in the XBP1u AP) in Figure 6B. [file elife-46267-supp2.docx]

**Supplementary Table 2**

N-glycosylation acceptor site

XBP1u arrest peptide (21 residues)

Flanking GGPG….GPGG tetrapeptides

H segment

Linker

Amino acid sequences of constructs representing the red curve in Figure 6B (mutation S255A in the XBP1u arrest peptide)

L69:

MANMFALILVIATLVTGILWCVDKFFFAPKRRERQAAAQAAAGDSLDKATLKKVAPKPGWLETGASVFPVLAIVLIVRSFIYEPFQIPSGSMMPTLNSTDFILVEKFAYGIKDPIYQKTLIETGHPKRGDIVVFKYPEDPKLDYIKRAVGLPGDKVTYDPVSKELTIQPGCSSGQACENALPVTYSNVEPSDFVQTFSRRNGGEATSGFFEVPKQETKENGIRLSETSGGPGAAAALALALALALALAAAAGPGGTWIVPPGQYFMMGDNRDNSADSRYWGFVPEANLVGRATAGDPVPYQPPFLCQWGRHQPAWKPLMNGSSDKQEGEWPTGLRLSRIGGIH*

L63:

MANMFALILVIATLVTGILWCVDKFFFAPKRRERQAAAQAAAGDSLDKATLKKVAPKPGWLETGASVFPVLAIVLIVRSFIYEPFQIPSGSMMPTLNSTDFILVEKFAYGIKDPIYQKTLIETGHPKRGDIVVFKYPEDPKLDYIKRAVGLPGDKVTYDPVSKELTIQPGCSSGQACENALPVTYSNVEPSDFVQTFSRRNGGEATSGFFEVPKQETKENGIRLSETSGGPGAAAALALALALALALAAAAGPGGGQYFMMGDNRDNSADSRYWGFVPEANLVGRATAGDPVP YQPPFLCQWGRHQPAWKPLMNGSSDKQEGEWPTGLRLSRIGGIH*

L61:

MANMFALILVIATLVTGILWCVDKFFFAPKRRERQAAAQAAAGDSLDKATLKKVAPKPGWLETGASVFPVLAIVLIVRSFIYEPFQIPSGSMMPTLNSTDFILVEKFAYGIKDPIYQKTLIETGHPKRGDIVVFKYPEDPKLDYIKRAVGLPGDKVTYDPVSKELTIQPGCSSGQACENALPVTYSNVEPSDFVQTFSRRNGGEATSGFFEVPKQETKENGIRLSETSGGPGAAAALALALALALALAAAAGPGGYFMMGDNRDNSADSRYWGFVPEANLVGRATAGDPVPYQPPFLCQWGRHQPAWKPLMNGSSDKQEGEWPTGLRLSRIGGIH*

L59:

MANMFALILVIATLVTGILWCVDKFFFAPKRRERQAAAQAAAGDSLDKATLKKVAPKPGWLETGASVFPVLAIVLIVRSFIYEPFQIPSGSMMPTLNSTDFILVEKFAYGIKDPIYQKTLIETGHPKRGDIVVFKYPEDPKLDYIKRAVGLPGDKVTYDPVSKELTIQPGCSSGQACENALPVTYSNVEPSDFVQTFSRRNGGEATSGFFEVPKQETKENGIRLSETSGGPGAAAALALALALALALAAAAGPGGMMGDNRDNSADSRYWGFVPEANLVGRATAGDPVP YQPPFLCQWGRHQPAWKPLMNGSSDKQEGEWPTGLRLSRIGGIH*

L56:

MANMFALILVIATLVTGILWCVDKFFFAPKRRERQAAAQAAAGDSLDKATLKKVAPKPGWLETGASVFPVLAIVLIVRSFIYEPFQIPSGSMMPTLNSTDFILVEKFAYGIKDPIYQKTLIETGHPKRGDIVVFKYPEDPKLDYIKRAVGLPGDKVTYDPVSKELTIQPGCSSGQACENALPVTYSNVEPSDFVQTFSRRNGGEATSGFFEVPKQETKENGIRLSETSGGPGAAAALALALALALALAAAAGPGGDNRDNSADSRYWGFVPEANLVGRATAGDPVP YQPPFLCQWGRHQPAWKPLMNGSSDKQEGEWPTGLRLSRIGGIH*

L53:

MANMFALILVIATLVTGILWCVDKFFFAPKRRERQAAAQAAAGDSLDKATLKKVAPKPGWLETGASVFPVLAIVLIVRSFIYEPFQIPSGSMMPTLNSTDFILVEKFAYGIKDPIYQKTLIETGHPKRGDIVVFKYPEDPKLDYIKRAVGLPGDKVTYDPVSKELTIQPGCSSGQACENALPVTYSNVEPSDFVQTFSRRNGGEATSGFFEVPKQETKENGIRLSETSGGPGAAAALALALALALALAAAAGPGGDNSADSRYWGFVPEANLVGRATAGDPVP YQPPFLCQWGRHQPAWKPLMNGSSDKQEGEWPTGLRLSRIGGIH*

L51:

MANMFALILVIATLVTGILWCVDKFFFAPKRRERQAAAQAAAGDSLDKATLKKVAPKPGWLETGASVFPVLAIVLIVRSFIYEPFQIPSGSMMPTLNSTDFILVEKFAYGIKDPIYQKTLIETGHPKRGDIVVFKYPEDPKLDYIKRAVGLPGDKVTYDPVSKELTIQPGCSSGQACENALPVTYSNVEPSDFVQTFSRRNGGEATSGFFEVPKQETKENGIRLSETSGGPGAAAALALALALALALAAAAGPGGSADSRYWGFVPEANLVGRATAGDPVPYQPPFLCQWGRHQPAWKPLMNGSSDKQEGEWPTGLRLSRIGGIH*

L48:

MANMFALILVIATLVTGILWCVDKFFFAPKRRERQAAAQAAAGDSLDKATLKKVAPKPGWLETGASVFPVLAIVLIVRSFIYEPFQIPSGSMMPTLNSTDFILVEKFAYGIKDPIYQKTLIETGHPKRGDIVVFKYPEDPKLDYIKRAVGLPGDKVTYDPVSKELTIQPGCSSGQACENALPVTYSNVEPSDFVQTFSRRNGGEATSGFFEVPKQETKENGIRLSETSGGPGAAAALALALALALALAAAAGPGGSRYWGFVPEANLVGRATAGDPVPYQPPFLCQWGRHQPAWKPLMNGSSDKQEGEWPTGLRLSRIGGIH*

L46:

MANMFALILVIATLVTGILWCVDKFFFAPKRRERQAAAQAAAGDSLDKATLKKVAPKPGWLETGASVFPVLAIVLIVRSFIYEPFQIPSGSMMPTLNSTDFILVEKFAYGIKDPIYQKTLIETGHPKRGDIVVFKYPEDPKLDYIKRAVGLPGDKVTYDPVSKELTIQPGCSSGQACENALPVTYSNVEPSDFVQTFSRRNGGEATSGFFEVPKQETKENGIRLSETSGGPGAAAALALALALALALAAAAGPGGYWGFVPEANLVGRATAGDPVPYQPPFLCQWGRHQPAWKPLMNGSSDKQEGEWPTGLRLSRIGGIH*

L45:

MANMFALILVIATLVTGILWCVDKFFFAPKRRERQAAAQAAAGDSLDKATLKKVAPKPGWLETGASVFPVLAIVLIVRSFIYEPFQIPSGSMMPTLNSTDFILVEKFAYGIKDPIYQKTLIETGHPKRGDIVVFKYPEDPKLDYIKRAVGLPGDKVTYDPVSKELTIQPGCSSGQACENALPVTYSNVEPSDFVQTFSRRNGGEATSGFFEVPKQETKENGIRLSETSGGPGAAAALALALALALALAAAAGPGGWGFVPEANLVGRATAGDPVPYQPPFLCQWGRHQPAWKPLMNGSSDKQEGEWPTGLRLSRIGGIH*

L43:

MANMFALILVIATLVTGILWCVDKFFFAPKRRERQAAAQAAAGDSLDKATLKKVAPKPGWLETGASVFPVLAIVLIVRSFIYEPFQIPSGSMMPTLNSTDFILVEKFAYGIKDPIYQKTLIETGHPKRGDIVVFKYPEDPKLDYIKRAVGLPGDKVTYDPVSKELTIQPGCSSGQACENALPVTYSNVEPSDFVQTFSRRNGGEATSGFFEVPKQETKENGIRLSETSGGPGAAAALALALALALALAAAAGPGGFVPEANLVGRATAGDPVPYQPPFLCQWGRHQPAWKPLMNGSSDKQEGEWPTGLRLSRIGGIH*

L42:

MANMFALILVIATLVTGILWCVDKFFFAPKRRERQAAAQAAAGDSLDKATLKKVAPKPGWLETGASVFPVLAIVLIVRSFIYEPFQIPSGSMMPTLNSTDFILVEKFAYGIKDPIYQKTLIETGHPKRGDIVVFKYPEDPKLDYIKRAVGLPGDKVTYDPVSKELTIQPGCSSGQACENALPVTYSNVEPSDFVQTFSRRNGGEATSGFFEVPKQETKENGIRLSETSGGPGAAAALALALALALALAAAAGPGGVPEANLVGRATAGDPVPYQPPFLCQWGRHQPAWKPLMNGSSDKQEGEWPTGLRLSRIGGIH*

L41:

MANMFALILVIATLVTGILWCVDKFFFAPKRRERQAAAQAAAGDSLDKATLKKVAPKPGWLETGASVFPVLAIVLIVRSFIYEPFQIPSGSMMPTLNSTDFILVEKFAYGIKDPIYQKTLIETGHPKRGDIVVFKYPEDPKLDYIKRAVGLPGDKVTYDPVSKELTIQPGCSSGQACENALPVTYSNVEPSDFVQTFSRRNGGEATSGFFEVPKQETKENGIRLSETSGGPGAAAALALALALALALAAAAGPGGPEANLVGRATAGDPVPYQPPFLCQWGRHQPAWKPLMNGSSDKQEGEWPTGLRLSRIGGIH*

L39:

MANMFALILVIATLVTGILWCVDKFFFAPKRRERQAAAQAAAGDSLDKATLKKVAPKPGWLETGASVFPVLAIVLIVRSFIYEPFQIPSGSMMPTLNSTDFILVEKFAYGIKDPIYQKTLIETGHPKRGDIVVFKYPEDPKLDYIKRAVGLPGDKVTYDPVSKELTIQPGCSSGQACENALPVTYSNVEPSDFVQTFSRRNGGEATSGFFEVPKQETKENGIRLSETSGGPGAAAALALALALALALAAAAGPGGANLVGRATAGDPVPYQPPFLCQWGRHQPAWKPLMNGSSDKQEGEWPTGLRLSRIGGIH*

L38:

MANMFALILVIATLVTGILWCVDKFFFAPKRRERQAAAQAAAGDSLDKATLKKVAPKPGWLETGASVFPVLAIVLIVRSFIYEPFQIPSGSMMPTLNSTDFILVEKFAYGIKDPIYQKTLIETGHPKRGDIVVFKYPEDPKLDYIKRAVGLPGDKVTYDPVSKELTIQPGCSSGQACENALPVTYSNVEPSDFVQTFSRRNGGEATSGFFEVPKQETKENGIRLSETSGGPGAAAALALALALALALAAAAGPGGNLVGRATAGDPVPYQPPFLCQWGRHQPAWKPLMNGSSDKQEGEWPTGLRLSRIGGIH*

L36:

MANMFALILVIATLVTGILWCVDKFFFAPKRRERQAAAQAAAGDSLDKATLKKVAPKPGWLETGASVFPVLAIVLIVRSFIYEPFQIPSGSMMPTLNSTDFILVEKFAYGIKDPIYQKTLIETGHPKRGDIVVFKYPEDPKLDYIKRAVGLPGDKVTYDPVSKELTIQPGCSSGQACENALPVTYSNVEPSDFVQTFSRRNGGEATSGFFEVPKQETKENGIRLSETSGGPGAAAALALALALALALAAAAGPGGVGRATAGDPVPYQPPFLCQWGRHQPAWKPLMNGSSDKQEGEWPTGLRLSRIGGIH*

L34:

MANMFALILVIATLVTGILWCVDKFFFAPKRRERQAAAQAAAGDSLDKATLKKVAPKPGWLETGASVFPVLAIVLIVRSFIYEPFQIPSGSMMPTLNSTDFILVEKFAYGIKDPIYQKTLIETGHPKRGDIVVFKYPEDPKLDYIKRAVGLPGDKVTYDPVSKELTIQPGCSSGQACENALPVTYSNVEPSDFVQTFSRRNGGEATSGFFEVPKQETKENGIRLSETSGGPGAAAALALALALALALAAAAGPGGRATAGDPVPYQPPFLCQWGRHQPAWKPLMNGSSDKQEGEWPTGLRLSRIGGIH*

L33:

MANMFALILVIATLVTGILWCVDKFFFAPKRRERQAAAQAAAGDSLDKATLKKVAPKPGWLETGASVFPVLAIVLIVRSFIYEPFQIPSGSMMPTLNSTDFILVEKFAYGIKDPIYQKTLIETGHPKRGDIVVFKYPEDPKLDYIKRAVGLPGDKVTYDPVSKELTIQPGCSSGQACENALPVTYSNVEPSDFVQTFSRRNGGEATSGFFEVPKQETKENGIRLSETSGGPGAAAALALALALALALAAAAGPGGATAGDPVPYQPPFLCQWGRHQPAWKPLMNGSSDKQEGEWPTGLRLSRIGGIH*

L31:

MANMFALILVIATLVTGILWCVDKFFFAPKRRERQAAAQAAAGDSLDKATLKKVAPKPGWLETGASVFPVLAIVLIVRSFIYEPFQIPSGSMMPTLNSTDFILVEKFAYGIKDPIYQKTLIETGHPKRGDIVVFKYPEDPKLDYIKRAVGLPGDKVTYDPVSKELTIQPGCSSGQACENALPVTYSNVEPSDFVQTFSRRNGGEATSGFFEVPKQETKENGIRLSETSGGPGAAAALALALALALALAAAAGPGGAGDPVPYQPPFLCQWGRHQPAWKPLMNGSSDKQEGEWPTGLRLSRIGGIH*

L30:

MANMFALILVIATLVTGILWCVDKFFFAPKRRERQAAAQAAAGDSLDKATLKKVAPKPGWLETGASVFPVLAIVLIVRSFIYEPFQIPSGSMMPTLNSTDFILVEKFAYGIKDPIYQKTLIETGHPKRGDIVVFKYPEDPKLDYIKRAVGLPGDKVTYDPVSKELTIQPGCSSGQACENALPVTYSNVEPSDFVQTFSRRNGGEATSGFFEVPKQETKENGIRLSETSGGPGAAAALALALALALALAAAAGPGGGDPVPYQPPFLCQWGRHQPAWKPLMNGSSDKQEGEWPTGLRLSRIGGIH*

L29:

MANMFALILVIATLVTGILWCVDKFFFAPKRRERQAAAQAAAGDSLDKATLKKVAPKPGWLETGASVFPVLAIVLIVRSFIYEPFQIPSGSMMPTLNSTDFILVEKFAYGIKDPIYQKTLIETGHPKRGDIVVFKYPEDPKLDYIKRAVGLPGDKVTYDPVSKELTIQPGCSSGQACENALPVTYSNVEPSDFVQTFSRRNGGEATSGFFEVPKQETKENGIRLSETSGGPGAAAALALALALALALAAAAGPGGDPVPYQPPFLCQWGRHQPAWKPLMNGSSDKQEGEWPTGLRLSRIGGIH*

L25:

MANMFALILVIATLVTGILWCVDKFFFAPKRRERQAAAQAAAGDSLDKATLKKVAPKPGWLETGASVFPVLAIVLIVRSFIYEPFQIPSGSMMPTLNSTDFILVEKFAYGIKDPIYQKTLIETGHPKRGDIVVFKYPEDPKLDYIKRAVGLPGDKVTYDPVSKELTIQPGCSSGQACENALPVTYSNVEPSDFVQTFSRRNGGEATSGFFEVPKQETKENGIRLSETSGGPGAAAALALALALALALAAAADPVPYQPPFLCQWGRHQPAWKPLMNGSSDKQEGEWPTGLRLSRIGGIH*

Amino acid sequence list of constructs representing the blue curve in Figure. 6B (mutations P254C and S255A in the XBP1u arrest peptide)

L69:

MANMFALILVIATLVTGILWCVDKFFFAPKRRERQAAAQAAAGDSLDKATLKKVAPKPGWLETGASVFPVLAIVLIVRSFIYEPFQIPSGSMMPTLNSTDFILVEKFAYGIKDPIYQKTLIETGHPKRGDIVVFKYPEDPKLDYIKRAVGLPGDKVTYDPVSKELTIQPGCSSGQACENALPVTYSNVEPSDFVQTFSRRNGGEATSGFFEVPKQETKENGIRLSETSGGPGAAAALALALALALALAAAAGPGGTWIVPPGQYFMMGDNRDNSADSRYWG FVPEANLVGRATAGDPVPYQPPFLCQWGRHQCAWKPLMNGSSDKQEGEWPTGLRLSRIGGIH*

L63:

MANMFALILVIATLVTGILWCVDKFFFAPKRRERQAAAQAAAGDSLDKATLKKVAPKPGWLETGASVFPVLAIVLIVRSFIYEPFQIPSGSMMPTLNSTDFILVEKFAYGIKDPIYQKTLIETGHPKRGDIVVFKYPEDPKLDYIKRAVGLPGDKVTYDPVSKELTIQPGCSSGQACENALPVTYSNVEPSDFVQTFSRRNGGEATSGFFEVPKQETKENGIRLSETSGGPGAAAALALALALALALAAAAGPGGGQYFMMGDNRDNSADSRYWGFVPEANLVGRATAGDPVP YQPPFLCQWGRHQCAWKPLMNGSSDKQEGEWPTGLRLSRIGGIH*

L61:

MANMFALILVIATLVTGILWCVDKFFFAPKRRERQAAAQAAAGDSLDKATLKKVAPKPGWLETGASVFPVLAIVLIVRSFIYEPFQIPSGSMMPTLNSTDFILVEKFAYGIKDPIYQKTLIETGHPKRGDIVVFKYPEDPKLDYIKRAVGLPGDKVTYDPVSKELTIQPGCSSGQACENALPVTYSNVEPSDFVQTFSRRNGGEATSGFFEVPKQETKENGIRLSETSGGPGAAAALALALALALALAAAAGPGGYFMMGDNRDNSADSRYWGFVPEANLVGRATAGDPVPYQPPFLCQWGRHQCAWKPLMNGSSDKQEGEWPTGLRLSRIGGIH*

L59:

MANMFALILVIATLVTGILWCVDKFFFAPKRRERQAAAQAAAGDSLDKATLKKVAPKPGWLETGASVFPVLAIVLIVRSFIYEPFQIPSGSMMPTLNSTDFILVEKFAYGIKDPIYQKTLIETGHPKRGDIVVFKYPEDPKLDYIKRAVGLPGDKVTYDPVSKELTIQPGCSSGQACENALPVTYSNVEPSDFVQTFSRRNGGEATSGFFEVPKQETKENGIRLSETSGGPGAAAALALALALALALAAAAGPGGMMGDNRDNSADSRYWGFVPEANLVGRATAGDPVP YQPPFLCQWGRHQCAWKPLMNGSSDKQEGEWPTGLRLSRIGGIH*

L56:

MANMFALILVIATLVTGILWCVDKFFFAPKRRERQAAAQAAAGDSLDKATLKKVAPKPGWLETGASVFPVLAIVLIVRSFIYEPFQIPSGSMMPTLNSTDFILVEKFAYGIKDPIYQKTLIETGHPKRGDIVVFKYPEDPKLDYIKRAVGLPGDKVTYDPVSKELTIQPGCSSGQACENALPVTYSNVEPSDFVQTFSRRNGGEATSGFFEVPKQETKENGIRLSETSGGPGAAAALALALALALALAAAAGPGGDNRDNSADSRYWGFVPEANLVGRATAGDPVP YQPPFLCQWGRHQCAWKPLMNGSSDKQEGEWPTGLRLSRIGGIH*

L53:

MANMFALILVIATLVTGILWCVDKFFFAPKRRERQAAAQAAAGDSLDKATLKKVAPKPGWLETGASVFPVLAIVLIVRSFIYEPFQIPSGSMMPTLNSTDFILVEKFAYGIKDPIYQKTLIETGHPKRGDIVVFKYPEDPKLDYIKRAVGLPGDKVTYDPVSKELTIQPGCSSGQACENALPVTYSNVEPSDFVQTFSRRNGGEATSGFFEVPKQETKENGIRLSETSGGPGAAAALALALALALALAAAAGPGGDNSADSRYWGFVPEANLVGRATAGDPVP YQPPFLCQWGRHQCAWKPLMNGSSDKQEGEWPTGLRLSRIGGIH*

L51:

MANMFALILVIATLVTGILWCVDKFFFAPKRRERQAAAQAAAGDSLDKATLKKVAPKPGWLETGASVFPVLAIVLIVRSFIYEPFQIPSGSMMPTLNSTDFILVEKFAYGIKDPIYQKTLIETGHPKRGDIVVFKYPEDPKLDYIKRAVGLPGDKVTYDPVSKELTIQPGCSSGQACENALPVTYSNVEPSDFVQTFSRRNGGEATSGFFEVPKQETKENGIRLSETSGGPGAAAALALALALALALAAAAGPGGSADSRYWGFVPEANLVGRATAGDPVPYQPPFLCQWGRHQCAWKPLMNGSSDKQEGEWPTGLRLSRIGGIH*

L48:

MANMFALILVIATLVTGILWCVDKFFFAPKRRERQAAAQAAAGDSLDKATLKKVAPKPGWLETGASVFPVLAIVLIVRSFIYEPFQIPSGSMMPTLNSTDFILVEKFAYGIKDPIYQKTLIETGHPKRGDIVVFKYPEDPKLDYIKRAVGLPGDKVTYDPVSKELTIQPGCSSGQACENALPVTYSNVEPSDFVQTFSRRNGGEATSGFFEVPKQETKENGIRLSETSGGPGAAAALALALALALALAAAAGPGGSRYWGFVPEANLVGRATAGDPVPYQPPFLCQWGRHQCAWKPLMNGSSDKQEGEWPTGLRLSRIGGIH*

L46:

MANMFALILVIATLVTGILWCVDKFFFAPKRRERQAAAQAAAGDSLDKATLKKVAPKPGWLETGASVFPVLAIVLIVRSFIYEPFQIPSGSMMPTLNSTDFILVEKFAYGIKDPIYQKTLIETGHPKRGDIVVFKYPEDPKLDYIKRAVGLPGDKVTYDPVSKELTIQPGCSSGQACENALPVTYSNVEPSDFVQTFSRRNGGEATSGFFEVPKQETKENGIRLSETSGGPGAAAALALALALALALAAAAGPGGYWGFVPEANLVGRATAGDPVPYQPPFLCQWGRHQCAWKPLMNGSSDKQEGEWPTGLRLSRIGGIH*

L45:

MANMFALILVIATLVTGILWCVDKFFFAPKRRERQAAAQAAAGDSLDKATLKKVAPKPGWLETGASVFPVLAIVLIVRSFIYEPFQIPSGSMMPTLNSTDFILVEKFAYGIKDPIYQKTLIETGHPKRGDIVVFKYPEDPKLDYIKRAVGLPGDKVTYDPVSKELTIQPGCSSGQACENALPVTYSNVEPSDFVQTFSRRNGGEATSGFFEVPKQETKENGIRLSETSGGPGAAAALALALALALALAAAAGPGGWGFVPEANLVGRATAGDPVPYQPPFLCQWGRHQCAWKPLMNGSSDKQEGEWPTGLRLSRIGGIH*

L43:

MANMFALILVIATLVTGILWCVDKFFFAPKRRERQAAAQAAAGDSLDKATLKKVAPKPGWLETGASVFPVLAIVLIVRSFIYEPFQIPSGSMMPTLNSTDFILVEKFAYGIKDPIYQKTLIETGHPKRGDIVVFKYPEDPKLDYIKRAVGLPGDKVTYDPVSKELTIQPGCSSGQACENALPVTYSNVEPSDFVQTFSRRNGGEATSGFFEVPKQETKENGIRLSETSGGPGAAAALALALALALALAAAAGPGGFVPEANLVGRATAGDPVPYQPPFLCQWGRHQCAWKPLMNGSSDKQEGEWPTGLRLSRIGGIH*

L42:

MANMFALILVIATLVTGILWCVDKFFFAPKRRERQAAAQAAAGDSLDKATLKKVAPKPGWLETGASVFPVLAIVLIVRSFIYEPFQIPSGSMMPTLNSTDFILVEKFAYGIKDPIYQKTLIETGHPKRGDIVVFKYPEDPKLDYIKRAVGLPGDKVTYDPVSKELTIQPGCSSGQACENALPVTYSNVEPSDFVQTFSRRNGGEATSGFFEVPKQETKENGIRLSETSGGPGAAAALALALALALALAAAAGPGGVPEANLVGRATAGDPVPYQPPFLCQWGRHQCAWKPLMNGSSDKQEGEWPTGLRLSRIGGIH*

L41:

MANMFALILVIATLVTGILWCVDKFFFAPKRRERQAAAQAAAGDSLDKATLKKVAPKPGWLETGASVFPVLAIVLIVRSFIYEPFQIPSGSMMPTLNSTDFILVEKFAYGIKDPIYQKTLIETGHPKRGDIVVFKYPEDPKLDYIKRAVGLPGDKVTYDPVSKELTIQPGCSSGQACENALPVTYSNVEPSDFVQTFSRRNGGEATSGFFEVPKQETKENGIRLSETSGGPGAAAALALALALALALAAAAGPGGPEANLVGRATAGDPVPYQPPFLCQWGRHQCAWKPLMNGSSDKQEGEWPTGLRLSRIGGIH*

L39:

MANMFALILVIATLVTGILWCVDKFFFAPKRRERQAAAQAAAGDSLDKATLKKVAPKPGWLETGASVFPVLAIVLIVRSFIYEPFQIPSGSMMPTLNSTDFILVEKFAYGIKDPIYQKTLIETGHPKRGDIVVFKYPEDPKLDYIKRAVGLPGDKVTYDPVSKELTIQPGCSSGQACENALPVTYSNVEPSDFVQTFSRRNGGEATSGFFEVPKQETKENGIRLSETSGGPGAAAALALALALALALAAAAGPGGANLVGRATAGDPVPYQPPFLCQWGRHQCAWKPLMNGSSDKQEGEWPTGLRLSRIGGIH*

L38:

MANMFALILVIATLVTGILWCVDKFFFAPKRRERQAAAQAAAGDSLDKATLKKVAPKPGWLETGASVFPVLAIVLIVRSFIYEPFQIPSGSMMPTLNSTDFILVEKFAYGIKDPIYQKTLIETGHPKRGDIVVFKYPEDPKLDYIKRAVGLPGDKVTYDPVSKELTIQPGCSSGQACENALPVTYSNVEPSDFVQTFSRRNGGEATSGFFEVPKQETKENGIRLSETSGGPGAAAALALALALALALAAAAGPGGNLVGRATAGDPVPYQPPFLCQWGRHQCAWKPLMNGSSDKQEGEWPTGLRLSRIGGIH*

L36:

MANMFALILVIATLVTGILWCVDKFFFAPKRRERQAAAQAAAGDSLDKATLKKVAPKPGWLETGASVFPVLAIVLIVRSFIYEPFQIPSGSMMPTLNSTDFILVEKFAYGIKDPIYQKTLIETGHPKRGDIVVFKYPEDPKLDYIKRAVGLPGDKVTYDPVSKELTIQPGCSSGQACENALPVTYSNVEPSDFVQTFSRRNGGEATSGFFEVPKQETKENGIRLSETSGGPGAAAALALALALALALAAAAGPGGVGRATAGDPVPYQPPFLCQWGRHQCAWKPLMNGSSDKQEGEWPTGLRLSRIGGIH*

L34:

MANMFALILVIATLVTGILWCVDKFFFAPKRRERQAAAQAAAGDSLDKATLKKVAPKPGWLETGASVFPVLAIVLIVRSFIYEPFQIPSGSMMPTLNSTDFILVEKFAYGIKDPIYQKTLIETGHPKRGDIVVFKYPEDPKLDYIKRAVGLPGDKVTYDPVSKELTIQPGCSSGQACENALPVTYSNVEPSDFVQTFSRRNGGEATSGFFEVPKQETKENGIRLSETSGGPGAAAALALALALALALAAAAGPGGRATAGDPVPYQPPFLCQWGRHQCAWKPLMNGSSDKQEGEWPTGLRLSRIGGIH*

L33:

MANMFALILVIATLVTGILWCVDKFFFAPKRRERQAAAQAAAGDSLDKATLKKVAPKPGWLETGASVFPVLAIVLIVRSFIYEPFQIPSGSMMPTLNSTDFILVEKFAYGIKDPIYQKTLIETGHPKRGDIVVFKYPEDPKLDYIKRAVGLPGDKVTYDPVSKELTIQPGCSSGQACENALPVTYSNVEPSDFVQTFSRRNGGEATSGFFEVPKQETKENGIRLSETSGGPGAAAALALALALALALAAAAGPGGATAGDPVPYQPPFLCQWGRHQCAWKPLMNGSSDKQEGEWPTGLRLSRIGGIH*

L31:

MANMFALILVIATLVTGILWCVDKFFFAPKRRERQAAAQAAAGDSLDKATLKKVAPKPGWLETGASVFPVLAIVLIVRSFIYEPFQIPSGSMMPTLNSTDFILVEKFAYGIKDPIYQKTLIETGHPKRGDIVVFKYPEDPKLDYIKRAVGLPGDKVTYDPVSKELTIQPGCSSGQACENALPVTYSNVEPSDFVQTFSRRNGGEATSGFFEVPKQETKENGIRLSETSGGPGAAAALALALALALALAAAAGPGGAGDPVPYQPPFLCQWGRHQCAWKPLMNGSSDKQEGEWPTGLRLSRIGGIH*

L30:

MANMFALILVIATLVTGILWCVDKFFFAPKRRERQAAAQAAAGDSLDKATLKKVAPKPGWLETGASVFPVLAIVLIVRSFIYEPFQIPSGSMMPTLNSTDFILVEKFAYGIKDPIYQKTLIETGHPKRGDIVVFKYPEDPKLDYIKRAVGLPGDKVTYDPVSKELTIQPGCSSGQACENALPVTYSNVEPSDFVQTFSRRNGGEATSGFFEVPKQETKENGIRLSETSGGPGAAAALALALALALALAAAAGPGGGDPVPYQPPFLCQWGRHQCAWKPLMNGSSDKQEGEWPTGLRLSRIGGIH*

L29:

MANMFALILVIATLVTGILWCVDKFFFAPKRRERQAAAQAAAGDSLDKATLKKVAPKPGWLETGASVFPVLAIVLIVRSFIYEPFQIPSGSMMPTLNSTDFILVEKFAYGIKDPIYQKTLIETGHPKRGDIVVFKYPEDPKLDYIKRAVGLPGDKVTYDPVSKELTIQPGCSSGQACENALPVTYSNVEPSDFVQTFSRRNGGEATSGFFEVPKQETKENGIRLSETSGGPGAAAALALALALALALAAAAGPGGDPVPYQPPFLCQWGRHQCAWKPLMNGSSDKQEGEWPTGLRLSRIGGIH*

L25:

MANMFALILVIATLVTGILWCVDKFFFAPKRRERQAAAQAAAGDSLDKATLKKVAPKPGWLETGASVFPVLAIVLIVRSFIYEPFQIPSGSMMPTLNSTDFILVEKFAYGIKDPIYQKTLIETGHPKRGDIVVFKYPEDPKLDYIKRAVGLPGDKVTYDPVSKELTIQPGCSSGQACENALPVTYSNVEPSDFVQTFSRRNGGEATSGFFEVPKQETKENGIRLSETSGGPGAAAALALALALALALAAAADPVPYQPPFLCQWGRHQCAWKPLMNGSSDKQEGEWPTGLRLSRIGGIH*
